# Is bulbospongiosus muscle botox injection safe and effective in treating lifelong premature ejaculation? Randomized controlled study

**DOI:** 10.1007/s00345-024-04899-1

**Published:** 2024-04-06

**Authors:** Hussein Shaher, Karem Noah, Mohamed Abdelzaher, Wael Kandil, Islam Saied Ahmed, Islam S Nouh

**Affiliations:** 1 Urology and Andrology Department, Faculty of Medicine, Benha University, Benha, Egypt; 2 Department of Urology, Benha University Hospital, Farid Nada St, Benha, Egypt

**Keywords:** Premature ejaculation, Botulinum-A toxin, Bulbospongiosus muscle

## Abstract

**Purpose:**

To evaluate the safety and efficacy of botulinum-A toxin injections into the bulbospongiosus muscle for cases of lifelong drug-resistant premature ejaculation (PE).

**Methods:**

Ninety-eight outpatients diagnosed with lifelong PE were randomly assigned to two groups: the botulinum-A toxin group comprising forty-nine patients and the placebo (saline) group also consisting of forty-nine patients. A 100 U botulinum-A toxin was diluted into 10 cc of saline, with 5 cc injected into one side of the muscle (botulinum-A toxin group) guided by ultrasound to distribute across most muscle fibers. The same technique was applied using the same volume of saline injected into the bulbospongiosus muscle. Intravaginal ejaculatory latency time (IELT), scores from the premature ejaculation profile (PEP), Premature Ejaculation Diagnostic Tool (PEDT), International Index of Erectile Function (IIEF), and recording of any complications were obtained. Follow-ups occurred at 1-, 3-, and 6-month post-procedure.

**Results:**

Cases receiving injections of botulinum-A toxin into the bulbospongiosus muscle showed notably extended intravaginal ejaculatory latency times compared to their initial performance after treatment. In addition, there were enhancements in PEP scores, and notably, no significant complications were reported. Conversely, the bilateral injection of saline into the bulbospongiosus muscle did not demonstrate any impact on ejaculation latencies.

**Conclusion:**

Our study demonstrated that the injection of botulinum-A toxin into the bulbospongiosus muscle can serve as a safe and effective option for treating PE. Nonetheless, its clinical application warrants further studies involving larger sample sizes and longer follow-up periods.

## Introduction

Early ejaculation is very prevalent in males, as seen by reports [1], which place its incidence between 20 and 30%. Male sexual dysfunction known as lifelong premature ejaculation (PE) is characterized by recurrent or consistent ejaculation occurring within 1 min of vaginal penetration. This syndrome may lead to negative personal consequences such as distress, irritation, frustration, and even a reluctance to engage in sexual intimacy [2]. Although the exact etiology of PE remains unknown, it is thought to stem from a neurological pathway [3].

It is hypothesized that pharmacotherapy should be the treatment of choice for those with chronic PE [4, 5]. Unfortunately, at this time, the US Food and Drug Administration (FDA) has not granted approval to any drugs for the treatment of PE. On the other hand, dapoxetine, an SSRI characterized by its short half-life, has been authorized on-demand in over 50 countries for the management of PE [6, 7].

Botulinum-A toxin inhibits neuronal transmission via intramuscular administration by selectively blocking the release of acetylcholine from nerve terminals [8, 9]. Botulinum-A toxin has been shown to be an efficient and safe treatment for two urological disorders, namely Detrusor sphincter dysynergia and neurogenic detrusor overactivity [10, 11]. Involved in the ejaculatory reflex, local injection of botulinum-A can suppresses muscular contractions in the bulbospongiosus muscle [12, 13]. The concept of prescribing botulinum-A toxin to inhibit stereotyped rhythmic contractions of the bulbospongiosus muscle as a permanent treatment for PE was initially proposed in 2010 [13].

The aim of this study was to assess the safety and efficacy of botulinum-A toxin injection into the bulbospongiosus muscle for treating lifelong drug-resistant PE.

## Methods

In this study, which was prospective, randomized, double-blind, and placebo-controlled, 98 individuals from Benha University Hospitals’ outpatient clinics participated between October 2022 and September 2023. These participants were split into two cohorts: the botox group comprising 49 cases and the saline (placebo) group with an equal number of 49 cases. Before the study commenced, all participants provided informed written consent in accordance with the Declaration of Helsinki. In addition, the study received approval from the local ethics committee at the Faculty of Medicine, Benha University, marked as ethically approved under “Ms 35-10-2022.”

Before the procedure, patients were assured and informed that BOTOX is a material under trial with no major complications. Inclusion criteria were heterosexually active men experiencing lifelong PE, aged 20–50, and having failed prior medical treatments (behavioral therapy, SSRIs, topical anesthetic agents). These patients were evaluated based on the intravaginal ejaculatory latency time (IELT) (less than 1 min) and International Index of Erectile Function (IIEF) (26 points or more) to ensure good erection status. Exclusion criteria include patients with erectile dysfunction (ED) (IIEF less than 26 points), previous urethral operations, prostatitis (LUTS), diabetes (DM), pelvic operations, neurological diseases, chronic psychological illness and antipsychotic medications. Each participant underwent a comprehensive medical and sexual history assessment.

Evaluation criteria consisted of the intravaginal ejaculatory latency time (IELT), where a normal duration ranged from 2.5 to 5 min; scores from the premature ejaculation profile (PEP) [14]; the Premature Ejaculation Diagnostic Tool (PEDT) [15], with scores of 11 or higher indicating common occurrence of PE, 9 or 10 indicating a “borderline” score, and 8 or lower suggesting the absence of PE; and erectile function assessed by the IIEF [16]. On the Visual Analog Scale (VAS), pain was rated from zero (indicating no discomfort) to ten (intolerable pain).

**Fig. 1:**
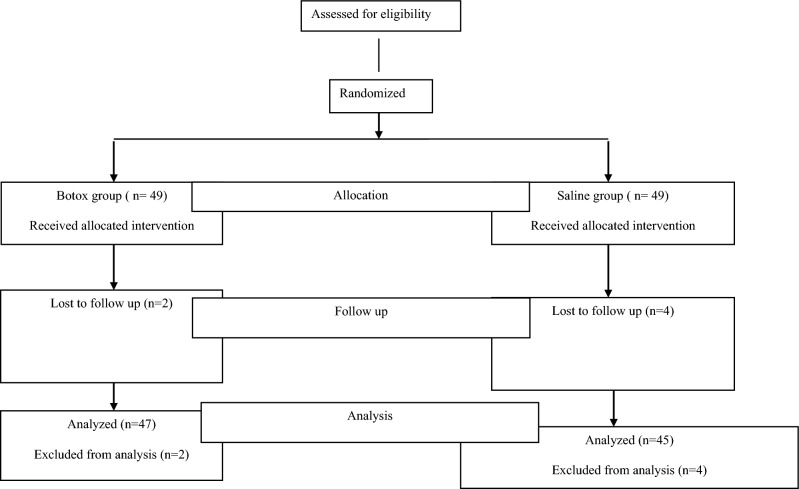
Flow diagram

Throughout the research, two participants in the botox group and four in the saline group were lost to follow-up and subsequently excluded from the study. Prior to the procedure, random allocation took place using sealed envelopes containing either BOTOX or saline assignments, which were opened exclusively before the commencement of the procedure (Fig. 1).

### The procedure

Patients were positioned in lithotomy position, the perineal area was disinfected with 70% alcohol, and the bulbospongiosus muscle was identified using ultrasound (6–13 Hz superficial probe with MAX depth 6 cm).

Botox group: Preparation for the BOTOX group involved diluting 100 U of botulinum-A toxin (Allergan, Ona botulinum toxin-A) into 10 mL of saline. Botox vial injection was administered as 5 mL into one side of the muscle, guided by ultrasound to distribute across most muscle fibers.

The placebo group: This group underwent a comparable procedure, differing only in the substance injected, as they were administered saline (10 mL) at identical injection sites.

To ensure blindness regarding the material used, syringes were concealed with plaster tape, maintaining this blindness for both the patient and the specialist involved. A specialist performed the injections while remaining unaware of the allocation.

### Follow-up

Patients underwent evaluations at 1, 3, and 6 months following the injection. Assessments included the IELT, scores from the PEP, the PEDT, changes in erection (IIEF), and the recording of any complications.

### Statistical analysis

The study employed Gpowersoft software version 3.1.7.9 to calculate the sample size based on Serefoglu et al.’s (2014) study, determining a minimum of 90 participants with a significance level of 0.05 and a type II error of 0.2. Subsequently, data collected were tabulated and statistically analyzed using SPSS software version 26.0 and Microsoft Excel 2016. Qualitative data were presented in numbers and percentages, while the Shapiro–Wilk test confirmed the normality of distribution. Quantitative data were described in terms of range, mean, standard deviation, median, and interquartile range (IQR), with a significance level set at 5%. Statistical tests included Friedman’s test for comparisons among non-parametric datasets across different time points and the Wilcoxon signed-rank test for analyzing related non-parametric samples. Interpretations were made based on significance levels: *P* ≥ 0.05 indicated non-significance (NS), *P* < 0.05 indicated significance (S), and *P* < 0.01 indicated high significance (HS).

## Results

As illustrated in Table 1, all baseline characteristics, encompassing age, BMI, medical history, and premature ejaculation duration, demonstrated comparability across the studied groups (*P* value >0.05). In the BOTOX group, the IELT exhibited a significant increase (70.00 ± 47.64 s) after 1 month, as it was tripled compared to the pre-injection score (35.79 ± 14.23 s), and a onefold increase after 3 months (60.36 ± 40.08 s). However, insignificant changes were observed at 6 months (42.64 ± 30.35 s) in comparison to the pre-injection score. Although these fore mentioned improvement were statistically significant, yet these outcomes were clinically insufficient; however, the enrolled patients were happy enough to finally experience some increase in their latency time after years of trying other treatment options specially SSRIs with no obvious improve. Despite of this short time improvement, many patients were motivated to repeat the injection another time as they found these injection cycles more cost-effective than the long-term chronically used medication they have tried before without satisfactory results specially that we were concerned to evaluate the cost-effective aspects of this trail as our facility is dealing with patients of lower socioeconomic levels. Conversely, patients who received saline injections showed only a 0.5% insignificant improvement, possibly attributable to the placebo effect, and reverted to baseline satisfaction scores during subsequent follow-up visits, as depicted in Table 2.

**Table 1:** Demographic characteristics among the studied groups

Parameters	Botox group (No. = 47)		Control group (No. = 45)		Test value	*P* value
	*N*	%	*N*	%		
*Age (years)*						
Mean ± SD	31.55 ± 5.3		31.82 ± 4.58		^Z^MWU = 0.380	0.703 (NS)
Median (IQR)	30 (27–37)		35			
Range	24–40		24–40			
*Medical history*						
No	42	89.4	40	88.9	*X*^2^ = 0.005	0.942 (NS)
HTN	5	10.6	5	11.1		
*BMI (kg/m*^*2*^*)*						
Mean ± SD	25.98 ± 3.41		26.76 ± 3.49		^Z^MWU = 1.104	0.270 (NS)
Median (IQR)	26 (23–29)		26 (24–29)			
Range	20–34		21–34			
Duration of PE (years)						
Mean ± SD	4.21 ± 3.0		3.59 ± 2.48		^Z^MWU = 0.862	0.388 (NS)
Median (IQR)	3 (2–6)		2 (2–5)			
Range	1–12		1–10			

*P* value >0.05: not significant (NS), *P* value <0.05 is statistically significant (S), *P* < 0.01 is highly significant (HS), SD: standard deviation, IQR: interquartile range, analysis done by Mann–Whitney *U* test and Chi-square test

**Table 2:** Comparison between the studied groups regarding changes in pretreatment and post-injection IELT, Premature Ejaculation Diagnostic Tools questionnaire, PEP and IIEF

	Botox group (No. = 47)						Control group (No. = 45)						Mann–Whitney *U* test	
	Mean ± SD	Median	IQR		Range		Mean ± SD	Median	IQR		Range		Test value	*P* value
*IELT (injection/s)*														
Pretreatment	35.79 ± 14.23	38	23	49	6	59	33.87 ± 14.15	35	24	47	6	59	0.692	0.489 (NS)
1 month post-injection	70.00 ± 47.64	50	35	112	7	175	34.76 ± 15.25	35	25	48	7	80	3.70	<0.001 (HS)
3 months post-injection	60.36 ± 40.08	48	35	100.0	6	150	33.73 ± 14.11	35	24	46	6	59	3.158	0.002 (HS)
6 months post-injection	42.64 ± 30.35	39	21	49.0	6	149	33.64 ± 13.94	35	24	46	6	59	0.868	0.386 (NS)
*P* value	*P*<0.001 (HS) *P*1 < 0.001, *P*2 < 0.001, *P*3 = 0.162						*P* = 0.008 (HS) *P*1 = 0.288, *P*2 = 0.270, *P*3 = 0.939							
*Premature Ejaculation Diagnostic Tools questionnaire*														
Pretreatment	16.11 ± 1.77	16.0	15.0	18.0	12.0	20.0	16.31 ± 1.50	16.0	15.0	17.0	14.0	20.0	0.561	0.575 (NS)
1 month post-injection	13.19 ± 3.84	14.0	9.0	16.0	8.0	20.0	16.11 ± 1.84	16.0	15.0	17.0	9.0	20.0	3.467	0.001 (HS)
3 months post-injection	14.23 ± 2.88	14.0	13.0	16.0	8.0	20.0	16.22 ± 1.52	16.0	15.0	17.0	14.0	20.0	3.753	<0.001(HS)
6 months post-injection	15.49 ± 2.87	16.0	14.0	18.0	8.0	20.0	16.36 ± 1.46	16.0	16.0	17.0	14.0	20.0	0.929	0.353 (NS)
*P* value	*P* < 0.001 *P*1 < 0.001, *P*2 = 0.027, *P*3 = 0.472						*P* = 0.300 (NS)							
*PEP*														
Pretreatment	5.49 ± 2.17	5.0	4.0	7.0	1.0	15.0	5.00 ± 1.43	5.0	4.0	6.0	1.0	8.0	1.159	0.246 (NS)
1 month post-injection	8.36 ± 4.20	7.0	5.0	12.0	1.0	17.0	5.16 ± 1.86	5.0	4.0	6.0	1.0	13.0	3.738	<0.001 (HS)
3 months post-injection	7.19 ± 3.43	7.0	5.0	8.0	1.0	17.0	5.00 ± 1.43	5.0	4.0	6.0	1.0	8.0	3.636	<0.001 (HS)
6 months post-injection	6.11 ± 3.27	5.0	4.0	7.0	1.0	17.0	5.00 ± 1.43	5.0	4.0	6.0	1.0	8.0	1.403	0.161 (NS)
*P* value	*P*<0.001 *P*1 < 0.001, *P*2 = 0.027, *P*3 = 0.472						*P* = 0.392 (NS)							
*IIEF*														
Pretreatment	27.40 ± 1.08	27.0	26.0	28.0	26.0	30.0	27.56 ± 1.01	28.0	27.0	28.0	26.0	29.0	0.742	0.458 (NS)
6 months post-injection	27.23 ± 1.45	27.0	26.0	28.0	23.0	30.0	27.49 ± 1.20	28.0	27.0	28.0	23.0	29.0	0.785	0.433 (NS)
*P* value	0.102 (NS)						0.317 (NS)							

*P* value >0.05: not significant (NS), *P* value <0.05 is statistically significant (S), *P* < 0.01 is highly significant (HS), SD: standard deviation, IQR: interquartile range, IELT: intravaginal ejaculatory latency time, PEP: premature ejaculation profile

*P*1: *P* value between pretreatment and 1 month post-injection, *P*2: *P* value between pretreatment and 3 months post-injection, *P*3: *P* value between pretreatment and 6 months post-injection

The scores from the PEDT questionnaire and the PEP significantly improved after 1 month (13.19 ± 3.84 and 8.36 ± 4.20) and also after 3 months ( 14.23 ± 2.88 and 7.19 ± 3.43) compared to the pre-injection score ( 16.11 ± 1.77 and 5.49 ± 2.17), respectively. However, there were no significant changes observed at 6 months (15.49 ± 2.87 and 6.11 ± 3.27) compared to the pre-injection score (16.11 ± 1.77 and 5.49 ± 2.17) for PEDT and PEP, respectively, as detailed in Table 2. Further analysis of the Botox effect revealed that 44.7% of patients showed improvement after 1 and 3 months, reducing to only 8.5% after 6 months, as indicated in Table 3. Regarding the IIEF score, no changes in erection were noted between pre- and post-injection IIEF (*P* value >0.05), as displayed in Table 2.

**Table 3:** Comparison between the studied groups regarding outcome

Parameters	Botox group (No. = 47)		Control group (No. = 45)		Chi-square test	
	*N*	%	*N*	%	Test value	*P* value
*VAS post-injection*						
Mean ± SD	4.51 ± 1.46		4.11 ± 1.43		^Z^MWU = 1.277	0.201 (NS)
Median (IQR)	5 (3–6)		4 (3–5)			
Range	2–7		2–7			
*Improvement*						
1 month post-injection	21	44.7	1	2.2	*X*^2^ = 23.58	<0.001 (HS)
3 months post-injection	21	44.7	0	0.0	*X*^2^ = 20.50	<0.001 (HS)
6 months post-injection	4	8.5	0	0.0	*X*^2^ = 2.219	0.136 (NS)
*Post-injection complications*						
No	43	91.5	45	100.0	*X*^2^ = 4.004	0.378^MC^ (NS)
Drippling	2	4.3	0	0.0		
Infection	1	2.1	0	0.0		
Pain	1	2.1	0	0.0		

*P* value >0.05: not significant (NS), *P* value <0.05 is statistically significant (S), *P* < 0.01 is highly significant (HS), analysis done by Chi-square test

The saline group did not experience significant changes in IELT, PEP, or the score from the PEDT in comparison to baseline values (Table 2). In terms of treatment-induced pain, there was no notable difference in the VAS score between the botox and control groups (4.51 ± 1.46 vs. 4.11 ± 1.43, respectively), Table 3.

Post-injection complications were reported in four cases (8.5%) of the study group. Among these, two patients (4.3%) experienced post-micturition dribbling, one developed an infection managed with appropriate antibiotics, and one patient required NSAIDs for 1 day due to pain, detailed in Table 3.

## Discussion

Premature ejaculation stands as one of the most prevalent sexual dysfunctions globally [17]. Around 30% of men between 18- and 59-years old report experiencing premature ejaculation issues, although some studies suggest a prevalence as high as 75% [18, 19]. Topical anesthetics applied to the penis have shown some success and offer an alternative to SSRIs, effectively avoiding potential systemic side effects [20]. SSRIs are typically the primary medical treatment for most premature ejaculation cases, even though they are commonly used off-label for treating both primary and secondary premature ejaculation cases [21]. As it is one of the theories proposed to explain the whole process of ejaculation, the primary role of bulbospongiosus and ischiocavernosus muscles were investigated as a part of a spinal cord reflex ending with rhythmic contractions of these muscles that facilitate semen propulsion throughout the urethra during the ejaculation phase [13, 22]. Botulinum-A toxin acts as a selective acetylcholine release blocker, impeding neural transmission upon injection into muscles and based on that, it has been used for years to treat a wide range of medical conditions related to muscle spasms and neurological insults such as blepharospasm, strabismus, detrusor overactivity, and detrusor sphincteric dyssynergia [13]. Based on that assumption, previous study was published to evaluate the potential role of BOTOX injection in theses muscles aiming to improve the ejaculation time [13]. Similarly, the hypothesis of BOTOX injection in bulbospongiosus muscles was proposed as a potential management option for patient with refractory PE [13, 23, 24].

Our study delves into the effect of BOTOX injections into the bulbospongiosus muscle in patients with treatment-resistant premature ejaculation. While animal studies have been conducted to assess the impact of BOTOX injections in this muscle on ejaculation latency [23, 24], there have been similar human studies limited by small group sizes and short-term outcomes [25]. For instance, Ongün et al. [23] reported significant improvements in ejaculatory latency in rats injected with botulinum toxin in the bulbospongiosus muscle, demonstrating longer ejaculation times compared to controls. Similar findings were observed by Serefoglu et al. [24], showing significantly longer ejaculatory latencies in rats treated with botulinum-A toxin.

Our recent study, a double-blind, randomized, placebo-controlled investigation guided by ultrasound during injection, demonstrated substantial improvement in IELT, PEP, and PEDT at 1- and 3-month post-BOTOX injection. However, there was no significant change observed at 6 months compared to pretreatment. Interestingly, only 0.5% of participants in the saline group exhibited negligible improvement, likely influenced by the placebo effect, reverting to baseline satisfaction scores in subsequent follow-up visits. Our findings align with Li et al.’s study [25], indicating longer IELT post-BOTOX injection compared to controls. In addition, our study revealed significant improvements in PEP and PEDT scores post-injection.

The effectiveness rate in our study after 1 month was 44.7%, akin to Li et al. [25], who demonstrated an effectiveness rate of 47.06%. However, our study showed a decline in effectiveness to 8% after 6 months, indicating a reduced effect over time. Regarding adverse reactions, Li et al. [25] reported complications in six cases (17.65%) in the trial group, including decreased erectile hardness and incomplete urination. Conversely, our study reported complications in four cases (8.5%) of the study population, such as post-micturition dribbling, infection managed with antibiotics, and transient pain requiring NSAIDs.

Nevertheless, limitations of this study, such as the small sample size, single-center approach, and short follow-up duration, need acknowledgment.

## Conclusion

Administering botulinum-A toxin into the bulbospongiosus muscle demonstrates both safety and efficacy as a treatment for lifelong PE. However, its clinical implementation requires additional exploration via studies that encompass larger sample sizes and more extended follow-up periods.

## Data Availability

Data available upon reasonable request from the authors.
